# Determinants of Urinary Incontinence and Subtypes Among the Elderly in Nursing Homes

**DOI:** 10.3389/fpubh.2021.788642

**Published:** 2021-12-06

**Authors:** Hongyan Tai, Shunying Liu, Haiqin Wang, Hongzhuan Tan

**Affiliations:** ^1^Clinical Nursing Teaching and Research Section, The Second Xiangya Hospital, Central South University, Changsha, China; ^2^Geriatric Department, The Second Xiangya Hospital, Central South University, Changsha, China; ^3^Xiangya School of Public Health, Central South University, Changsha, China

**Keywords:** urinary incontinence, geriatric, older adult, elderly population, prevalence, risk factor, nursing home, subtype

## Abstract

Urinary incontinence (UI) is a common problem among older adults. This study investigated the prevalence of UI in nursing home residents aged ≥75 years in China and examined potential risk factors associated with UI and its subtypes. Data were collected during face-to-face interviews using a general questionnaire, the International Consultation Incontinence Questionnaire Short-Form, and the Barthel Index. A total of 551 participants aged ≥75 years residing in Changsha city were enrolled from June to December 2018. The UI prevalence rate among nursing home residents aged ≥75 years was 24.3%. The most frequent subtype was mixed (M) UI (38.1%), followed by urge (U) UI (35.1%), stress (S) UI (11.9%), and other types (14.9%). In terms of severity, 57.5% had moderate UI, while 35.1% had mild and 7.5% had severe UI. Constipation, immobility, wheelchair use, cardiovascular disease (CVD), and pelvic or spinal surgery were significant risk factors for UI. Participants with a history of surgery had higher risks of SUI (odds ratio [OR] = 4.87, 95% confidence interval [CI]: 1.55–15.30) and UUI (OR = 1.97, 95% CI: 1.05–3.71), those who were immobile or used a wheelchair had higher rates of MUI (OR = 11.07, 95% CI: 4.19–29.28; OR = 3.36, 95% CI: 1.16–9.78) and other UI types (OR = 7.89, 95% CI: 1.99–31.30; OR = 14.90, 95% CI: 4.88–45.50), those with CVD had a higher rate of UUI (OR = 2.25, 95% CI: 1.17–4.34), and those with diabetes had a higher risk of UUI (OR = 2.250, 95% CI: 1.14–4.44). Use of oral antithrombotic agents increased UUI risk (OR = 4.98, 95% CI: 2.10–11.85) whereas sedative hypnotic drug use was associated with a higher risk of MUI (OR = 3.62, 95% CI: 1.25–10.45). Each UI subtype has distinct risk factors, and elderly residents of nursing homes with a history of CVD and pelvic or spinal surgery who experience constipation should be closely monitored. Reducing time spent in bed and engaging in active rehabilitation including walking and muscle strengthening may aid in UI prevention and treatment.

## Introduction

Urinary incontinence (UI) is defined by International Continence Society (ICS) as complaint of involuntary loss of urine (1). It's common in elderly patients and usually play a major role in independent person in the community or dependent person in the nursing home (2, 3). UI affects nearly 40% of women aged 80 years and older, 10–35% of older men, and up to 80% of long-term care residents and can severely impair an individual's quality of life (QoL) (4, 5) because of the associated hygiene and social problems (6). Therefore, healthcare providers need to demonstrate sensitivity in evaluating and discussing UI, particularly with older adults.

There are three major subtypes of UI—urge (U), stress (S), and mixed (M)—which have different risk factors and etiologies (7). UUI and SUI are the most common subtypes in older persons, while MUI is the combination of both types (8). UI is a risk factor for mortality in the elderly (9–11) and is closely related to declines in cognitive function and performance of activities of daily living (ADL) as well as age, obesity, diabetes, loss of independence, depression and anxiety levels, and agitation (8, 11–14). It is important to clarify the association between UI and cardiovascular risk factors through screening (14) to prevent the development of cardiovascular disease (CVD). However, there is little known about the prevalence of UI in the elderly population of China.

There are many factors that affect urinary incontinence in older adults, such as age, frailty, depression, neurologic conditions, cognitive impairment, mobility impairment, lower urinary tract symptoms (15–18), race, education, hypertension, smoking, diabetes, increased parity, higher body mass index, and oral hormone therapy, and radical prostatectomy for prostate cancer in men (15, 16, 19–21). However, in the very old population, whether there are more special factors of UI or not for this high-risk population, these are worthy of our consideration.

The aims of this study was to determine the prevalence of UI in the very old population (aged ≥ 75 years old) in nursing homes, examine potential risk factors associated with UI and its subtypes, which may provide evidence for further development of UI strategies for this high-risk populations.

## Materials and Methods

### Study Design

This nursing institution-based cross-sectional study was carried out with face-to-face surveys conducted between June and December 2018 among older adults residing in Changsha city, the capital of Hunan province, China. Changsha is located in the east of Hunan, with an area of 11,819.5 km^2^. Changsha comprises Furong, Tianxin, Yuelu, Kaifu, Yuhua, and Wangcheng districts; Changsha and Ningxiang counties; and Liuyang city. The study involved 20 of the 83 nursing homes in Changsha, with over 551 nursing home residents enrolled.

### Study Population

Older adults ≥75 years old who had resided for a minimum of 1 year in the study area with normal cognition and communication ability were eligible to participate. Mentally unstable nursing home residents with life-threatening diseases were excluded. The study was approved by the Medical Ethics Committee of Central South University. Written informed consent to participate in the study was provided by the participants or their legal guardian/next of kin.

### Study Size

The sample size was calculated using the formula n = μα^2^π (1–π)/δ^2^, where α is 0.05; π is the prevalence rate of UI, which was taken to be 25.0% from a previous study (22); and δ is 0.15 π. Based on this calculation, the minimum sample size for this study was determined as 512.

### Survey Instrument

A questionnaire for collecting data on general characteristics was used in the face-to-face interview. The data included age, nationality, marital status, occupation, source of income, education level, and medical history, among other characteristics. Height and weight were measured with the same ruler and electronic scales for each participant.

UI was assessed using the International Consultation Incontinence Questionnaire—Short Form (ICIQ-SF), which continues to be the most internationally used questionnaire and has been translated into over 60 languages (23). It comprises three scored items and an unscored self-diagnosis item to determine the prevalence, frequency, and severity of urinary leakage and its impact on QoL (24). The sum of scores for the three items ranging from 0 to 21, and higher scores indicating increased UI severity and greater impact on QoL. The scale has demonstrated high internal reliability in British patients at a urology clinic and in a community-based study (Cronbach's α = 0.95) (24, 25). Mild UI was defined as < 7 points; moderate UI as 7–14 points; and severe UI as >14 points.

ADL performance was significantly associated with UI (12), the Barthel Index (BI) was used to assess each individual's ADL performance (Cronbach's α = 0.93) (12). This 100-point clinical rating index includes 10 items related to self-care ability (i.e., bowels, bladder, grooming, toilet use, feeding, dressing, and bathing) and mobility (i.e., transfer, mobility, and stairs), with a higher score indicating a lower level of physical dependence. The Barthel index scores are classified as follows: 0–20 points: total dependency; 21–60 points: high-level dependency; 61–90 points: mid-level dependency; 91–99 points: low-level dependency; 100 points: total independence (26).

### Data Analysis

EpiData v3.1 (https://www.epidata.dk/index.htm) and SPSS v25.0 (IBM Corp., Armonk, NY, USA) were used for data management and analysis, respectively. Numerical variables are expressed as the mean ± standard deviation (SD) and categorical variables as frequency and percentage. Differences in frequency distributions between groups were assessed with Pearson χ^2^ tests, and determinants of UI and its subtypes were assessed using binary logistic regression (LR) models. For all tests, 2-tailed *p* < 0.05 were considered statistically significant.

## Results

### General Characteristics of Older Adults in Nursing Homes

Of the 551 study participants, 67.0% were female; 55.7% were 70–79 years old, 44.3% were >80 years old, and the mean age (±SD) was 84.16 (±4.84) years. In terms of education level, 37.2% of participants had completed middle or high school and almost 40% had >40 years of work experience. Most participants had been married; 30.5% were still married and 67.9% were widowed. In most cases, the source of income was retirement pension (89.5%), and only 14.5% of participants had a monthly income <2,000 yuan. In terms of functional status, 62.1% of participants were fully independent in ADL as measured by BI; 6.4% were impaired, and 4.5% were disabled. Based on body mass index (BMI), 22.9% of participants were overweight and 5.1% were obese (BMI ≥ 28 kg/m^2^) (Table 1).

**Table 1:** General characteristics of older adults in nursing homes.

**Characteristics**	**Category**	***N* (%)**
Gender	Male	182 (33.0)
	Female	369 (67.0)
Age	<80 years old	307 (55.7)
	≥80 years old	244 (44.3)
Occupation	Institution	208 (37.7)
	Enterprise/individual household	196 (35.6)
	Others	147 (26.7)
Education level	Primary school	168 (30.5)
	Middle and high school	205 (37.2)
	At least bachelor	178 (32.3)
Marriage	Single and divorce	10 (1.8)
	Couples	167 (30.3)
	Widowed	374 (67.9)
Economic source	Retirement pension	493 (89.5)
	Others	58 (10.5)
Month income	<2,000 yuan	80 (14.5)
	2,000–2,999 yuan	180 (32.7)
	3,000–3,999 yuan	121 (21.9)
	≥4,000 yuan	170 (30.9)
ADL score	≤ 40	25 (4.5)
	41–60	35 (6.4)
	61–99	149 (27.0)
	100	342 (62.1)
Body index mass (kg/m^2^)	<18.5	50 (9.1)
	18.5–23.9	347 (63.0)
	24–27.9	126 (22.9)
	≥28	28 (5.0)

### UI Prevalence in the Geriatric Population of Nursing Homes

We found 134 UI in all 551 participants, with a UI prevalence rate of 24.3%. Of which MUI accounted to 38.1%; UUI 35.1%; SUI 11.9%; and other types 14.9%; 57.5% of UI was moderate UI, 35.1 % was mild UI, and 7.5% had severe UI (Table 2).

**Table 2:** The constituent ratio of different UI among 134 UI patients.

**Characteristics**	***N***	**ratio (%)**
**Type**		
SUI	16	11.9
UUI	47	35.1
MUI	51	38.1
Other UI	20	14.9
**Severity**		
Mild UI	47	35.1
Moderate UI	77	57.5
Severe UI	10	7.5
**Total UI**	134	100.0

### General Characteristics of UI in the Geriatric Population

There was no difference in UI prevalence between males (22.5%) and females (25.2%) or between obese (23.5%) and non-obese (39.3%) participants. Anxiety and depression were associated with higher rates of UI (32.3%, χ^2^ = 7.39, *p* = 0.007) and other types of UI (6.5%, χ^2^ = 4.91, *p* = 0.027). In terms of functional status, immobile participants had a higher frequency of UI and MUI (75.0%, 45.0%), whereas those who could walk independently had a low rate of UI (19.9%) (χ^2^ = 51.82, *p* < 0.001). Mobility was a strong predictor of MUI and other UI types (χ^2^ = 36.68, *p* < 0.001, χ^2^ = 41.82, *p* < 0.001). Participants with a history of hypertension or urinary tract infection (UTI) had a higher rates of UI than those without this medical history (hypertension: 27.8% vs. 19.4%, χ^2^ = 5.11, *p* = 0.024; UTI: 53.8% vs. 23.6%, χ^2^ = 6.31, *p* = 0.012). Participants with constipation had higher rates of UI and MUI than those without constipation (UI: 32.7% vs. 20.7%, χ^2^ = 6.31, *p* = 0.012; MUI: 13.9% vs. 7.3%, χ^2^ = 6.15, *p* = 0.013). Participants with a history of CVD had a higher rate of UI and UUI (UI: 32.9% vs. 21.3%, χ^2^ = 7.67, *p* = 0.006; UUI: 16.1% vs. 5.9%, χ^2^ = 14.12, *p* < 0.001) and those with a history of surgery had a higher rate of UI (30.6% vs. 20.3%, χ^2^ = 7.51, *p* = 0.006), SUI (5.6% vs. 1.2%, χ^2^ = 8.86, *p* = 0.003), and UUI (11.6%, χ^2^ = 4.22, *p* = 0.040) than those without these in their medical history. Finally, participants who were taking oral antilipidemic and antithrombotic medications had higher rates of UI than those who were not taking these drugs (antilipidemics: 44.0%, χ^2^ = 5.51, *p* = 0.019; antithrombotics: 44.1%, χ^2^ = 5.60, *p* = 0.018) (Table 3).

**Table 3:** Prevalence rate of UI in different character of nursing homes (*n* = 551).

**Characteristics**	**Category**	***N*/total (%)**	***χ^2^* (*P)***
**UI**			
Gender	Male	41/182 (22.5)	0.474 (0.491)
	Female	93/369 (25.2)	
Obesity	No	123/523 (23.5)	3.590 (0.058)
	Yes	11/28 (39.3)	
Anxiety/depression	No	84/369 (21.2)	7.385 (0.007)
	Yes	50/155 (32.3)	
Constipation	No	80/386 (20.7)	9.047 (0.003)
	Yes	54/165 (32.7)	
Mobility	Immobility	15/20 (75.0)	51.819 (<0.001)
	Wheelchair use	15/24 (62.5)	
	Assisted walking	13/50 (26.0)	
	Independent walking	91/457 (19.9)	
Cardiovascular disease	No	87/408 (21.3)	7.666 (0.006)
	Yes	47/143 (32.9)	
Hypertension	No	44/227 (19.4)	5.111 (0.024)
	Yes	90/324 (27.8)	
Surgical history	No	68/335 (20.3)	7.507 (0.006)
	Yes	66/216 (30.6)	
Urinary tract infection	No	127/538 (23.6)	6.307 (0.012)
	Yes	7/13 (53.8)	
Antilipidemic medications	No	123/526 (23.4)	5.511 (0.019)
	Yes	11/25 (44.0)	
Antithrombotic medications	No	120/517 (23.2)	5.595 (0.018)
	Yes	14/34 (41.2)	
**SUI**			
Surgical history	No	4/335 (1.2)	8.860 (0.003)
	Yes	12/216 (5.6)	
**UUI**			
Cardiovascular disease	No	24/408 (5.9)	14.124 (<0.001)
	Yes	23/143 (16.1)	
Surgical history	No	22/335 (6.6)	4.220 (0.040)
	Yes	25/216 (11.6)	
Antilipidemic medications	No	40/526 (7.6)	12.724 (<0.001)
	Yes	7/25 (28.0)	
Antithrombotic medications	No	36/517 (7.0)	26.358 (<0.001)
	Yes	11/34 (32.4)	
Alcohol consumption	No	41/520 (7.9)	4.933 (0.026)
	Yes	6/31 (19.4)	
Diabetes	No	16/122 (13.1)	4.222 (0.040)
	Yes	31/429 (7.20)	
**MUI**			
Constipation	No	28/386 (7.3)	6.151 (0.013)
	Yes	23/165 (13.9)	
Mobility	Immobility	9/20 (45.0)	36.679 (<0.001)
	Wheelchair use	5/24 (20.8)	
	Assisted walking	3/50 (6.0)	
	Independent walking	34/457 (7.4)	
Age	<85 years old	36/307 (11.7)	5.038 (0.025)
	≥85 years old	15/244 (6.1)	
Sedative hypnotic drug	No	46/527 (8.7)	4.004 (0.045)
	Yes	5/24 (20.8)	
**Other UI**			
Anxiety/depression	No	10/396 (2.5)	4.909 (0.027)
	Yes	10/155 (6.5)	
Mobility	Immobility	3/20 (15.0)	41.820 (<0.001)
	Wheelchair use	6/24 (25.0)	
	Assisted walking	1/50 (2.0)	
	Independent walking	10/457 (2.2)	

### Factors Associated With UI and Its Subtypes

Binary LR was carried out to evaluate the association between UI, SUI, and other types of UI (dependent variable, dichotomized into UI vs. no UI, SUI vs. no SUI, and other UI vs. no other UI) and general characteristics of UI (covariates: anxiety/depression, constipation, mobility, CVD, hypertension, history of surgery, UTI, antilipidemic and antithrombotic medications). UI-related characteristics that were significant on Pearson χ^2^ tests were entered into the LR model by backward stepwise regression, with mobility as the last categorical covariate (independent walking; α In = 0.05, α Out = 0.10).

Binary LR was used to assess the independent association between UUI (dependent variable, dichotomized into UUI vs. no UUI) and general characteristics of UI and MUI (covariates: anxiety/depression, constipation, mobility, CVD, hypertension, history of surgery, UTI, antilipidemic and antithrombotic medicines, alcohol consumption, and diabetes). All UI- and UUI-related characteristics that were significant in Pearson χ^2^ tests were entered into model by backward stepwise regression, with mobility as the last categorical covariate (independent walk; probability for stepwise entry = 0.05, removal = 0.10). The binary LR model was used to assess the independent association between MUI (dependent variable, dichotomized into MUI vs. no MUI) and general characteristics of MUI (covariates: anxiety/depression, constipation, mobility, CVD, hypertension, surgical history, UTI, antilipidemic and antithrombotic medicines, age, and sedative/hypnotic drugs). All UI- and MUI-related characteristics that were significant in Pearson χ^2^ tests were entered into the model by backward stepwise regression, with mobility as the last categorical covariate (independent walking; probability for stepwise entry = 0.05, removal = 0.10). The multivariate-adjusted odds ratios (ORs) and their 95% confidence intervals (CIs) and *p*-values were calculated. All analyses met the goodness-of-fit criterion as determined with the Hosmer–Lemeshow tests.

The results showed that constipation, immobility, wheelchair use, CVD, and history of surgery were significant risk factors for UI (Table 3). Participants with a history of surgery had a higher risk of SUI (OR = 4.87, 95% CI: 1.55–15.30) and UUI (OR = 1.97, 95% CI: 1.05–3.71), and immobile and wheelchair-assisted older adults had a higher frequency of MUI (OR = 11.07, 95% CI: 4.19–29.28; OR = 3.36, 95% CI: 1.16–9.78) and other types of UI (OR = 7.89, 95% CI: 1.99–31.30; OR = 14.90, 95% CI: 4.88–45.50). Compared to participants with no history of CVD, those with CVD history reported a higher frequency of UUI (OR = 2.25, 95% CI: 1.17–4.34). Participants with diabetes were more likely to experience UUI than those without diabetes (OR = 2.250, 95% CI: 1.14–4.44). Use of oral antithrombotic drugs was associated with a higher risk for UUI (OR = 4.98, 95% CI: 2.10–11.85), and a history of sedative hypnotic drug use was associated with a higher risk of MUI (OR = 3.62, 95% CI: 1.25–10.45) (Table 4).

**Table 4:** Multivariate logistic regression model with UI and subtypes in the geriatric population of nursing homes.

**Variables**	**UI**	**SUI**	**UUI**	**MUI**	**OTHER**
	***OR (95%CI)/P***	***OR (95%CI)/P***	***OR (95%CI)/P***	***OR (95%CI)/P***	***OR (95%CI)/P***
Constipation (reference = No)	1.62 (1.03,2.55)/0.038				
Immobility (reference = Independent walk)	13.13 (4.44, 38.80)/ <0.001			11.07 (4.19, 29.28)/ <0.001	7.89 (1.99, 31.30)/0.003
Wheelchair Independent (reference = walk)	6.58 (2.70, 16.03)/ <0.001			3.36 (1.16, 9.78)/0.026	14.90 (4.88, 45.50)/ <0.001
Cardiovascular disease (reference = No)	2.15 (1.34, 3.43)/0.001		2.25 (1.17, 4.34)/0.015		
Surgical history (reference = No)	2.02 (1.31, 3.12)/0.001	4.87 (1.55, 15.30)/0.007	1.97 (1.05, 3.71)/0.035		
Antithrombotic drugs (reference = No)			4.98 (2.10, 11.86)/ <0.001		
Diabetes (reference = No)			2.25 (1.14, 4.44)/0.019		
Sedative hypnotic drug (reference = No)				3.62 (1.25, 10.45)/0.018	
**Constant**	0.035	0.002	0.017	0.187	0.022

## Discussion

In this study, the UI prevalence of nursing home residents aged ≥75 years was 24.3%, which is lower than that reported in other studies of individuals aged ≥65 years (4, 22). General good health, consciousness, and good cognitive ability may explain the lower rate in our cohort. Our results showed that CVD was a risk factor for UI and UUI, which was in line with previous findings that UI had a high prevalence among heart failure patients (27, 28). As bladder function is affected by many cardiovascular risk factors, UI is a possible consequence of metabolic syndrome (14). Water–sodium retention and impaired bladder function are associated with CVD, while diuretics used in CVD treatment may lead to nocturia and increase the occurrence of UI (29). Despite patients' perception that diuretics are unpleasant and make it difficult for them to leave their home, patients in one study were generally compliant with their medication regime; nonetheless, nearly half experienced urine leakage, and most found urgency and incontinence bothersome (28). The assessment and management of UI or UUI in patients with CVD warrant further exploration.

The relationship between mobility in ADL and UI and its subtypes was evaluated based on immobility, wheelchair dependence, and assisted and independent walking. Immobility and wheelchair dependence were found to be risk factors for UI, MUI, and other UI types in the study participants, which is in accordance with earlier observations (29, 30). The use of walking aids and activity training may reduce or prevent the occurrence of UI in the elderly (30, 31); thus, promoting walking ability may be effective in preventing UI in nursing home residents.

Constipation has been shown to increase the risk of UI in the elderly (29, 32), which is consistent with our findings. The anatomy and angle of the urethra may be altered with chronic constipation, leading to problems such as overactive bladder (OAB), urinary retention, and UI (32, 33); conversely, treatment of constipation may prevent UI.

In contrast to a previous study (34), we found no relationship between diabetes and UI in older adults; however, diabetes can lead to glycosuria and has been shown to be associated with UUI risk in multiple models. In one study, diabetes was associated with increased urination frequency and urine volume and thereby exacerbated UI and OAB by osmotic diuresis (35). Thus, stabilizing blood glucose level is a potential strategy for preventing UUI.

A history of pelvic or spinal surgery was an independent risk factor for UI and two subtypes (SUI and UUI). Damage to nerves or connective tissues near or in the bladder can occur during surgery (29), and radical pelvic dissection can result in direct and indirect injury to the pelvic plexuses, resulting in SUI and UUI (36). UI prevalence was reported to be higher among patients who had undergone spinal surgery (37).

Sedative hypnotic and antithrombotic drug use was identified as a determinant of MUI risk in our cohort. Insomnia is among the most common sleep disorders in the geriatric population (38), and the use of nighttime sedatives in this group may lead to nocturnal enuresis by inducing a deep sleep from which an individual fails to awaken in order to void (36). Antithrombotic drugs that inhibit platelet or coagulation factors are commonly prescribed drugs for preventing and treating cardiovascular disorder (39). Repeated low doses of aspirin can block arachidonic acid receptors and inhibit thromboxane A2 production by acetylating a serine residue near the narrow catalytic site of the cyclooxygenase (COX)-1 channel (40); and high doses of aspirin inhibit both COX-1 and COX-2, which have anti-inflammatory and analgesic effects (41). Oral aspirin potentially inhibits COX and decreases prostaglandin (PGE)2, a regulator of inflammation and metabolism (42) that acts through the G protein-coupled PGE2 receptors (EP) 1, EP2, EP3, and EP4. EP1 and EP3 activation in the detrusor muscle of the bladder induces muscle contraction, whereas EP4 activation causes muscle relaxation (43). Aspirin may target EP1, EP2, EP3, and EP4 to reduce bladder sphincter contraction and detrusor relaxation, leading to involuntary urine leakage, although the precise underlying mechanism remains to be determined.

Obesity was not a significant risk factor for UI in our cohort in the binary LR model, which is inconsistent with previous findings (14, 44). Obesity was previously reported as a risk factor for UI, although the observed trends are contradictory, with decreased SUI and increased MUI rates found to be associated with higher BMI (44). Older males with a reduction in strength but not increases in body or fat mass were linked to an increased frequency of UI (45). Changes in body composition including an increase and redistribution of fat mass occur in old age, and current BMI classification may not accurately reflect the associated physical risks in the elderly, who may require age-specific BMI cut-off points (46). Thus, BMI in itself should not be considered as an independent predictor of UI in the geriatric population but should be considered in the context of fat mass, muscle strength, or other indicators.

## Conclusion

In this study, we found that distinct factors contribute to the risk of different UI subtypes. Our results indicate that care providers in nursing homes should pay particular attention to residents with a history of CVD and pelvic or spinal surgery who are at risk of UI and may benefit from the treatment of constipation, less time spent in bed, and active training in walking and muscle strengthening. Further study is needed into the relationship between the use of antithrombotic drugs and UI, and age-specific BMI cut-off points in the elderly population must be established to determine how these factors influence UI risk.

## Data Availability Statement

The raw data supporting the conclusions of this article will be made available by the authors, without undue reservation.

## Ethics Statement

The studies involving human participants were reviewed and approved by Medical Ethics Committee of Zhejiang Hospital. The patients/participants provided their written informed consent to participate in this study.

## Author Contributions

HTan and HTai designed the study. SL and HW performed the experiments. HTai wrote the manuscript and analyzed the data. All authors contributed to the article and approved the submitted version.

## Funding

This work was supported by National key research and development plan named Research on the construction and evaluation of a comprehensive demonstration base for prevention and control of elderly syndrome (2020YFC2008606) and Nursing Research Fund of the Second Xiangya Hospital in 2018 (2018-YHL-33).

## Conflict of Interest

The authors declare that the research was conducted in the absence of any commercial or financial relationships that could be construed as a potential conflict of interest.

## Publisher's Note

All claims expressed in this article are solely those of the authors and do not necessarily represent those of their affiliated organizations, or those of the publisher, the editors and the reviewers. Any product that may be evaluated in this article, or claim that may be made by its manufacturer, is not guaranteed or endorsed by the publisher.
